# Depressive Symptoms in Individuals With Persistent Postconcussion Symptoms: A Systematic Review and Meta-Analysis

**DOI:** 10.1001/jamanetworkopen.2022.48453

**Published:** 2022-12-27

**Authors:** Maude Lambert, Elena Sheldrake, Audrey-Ann Deneault, Anne Wheeler, Matthew Burke, Shannon Scratch

**Affiliations:** 1 School of Psychology, University of Ottawa, Ottawa, Ontario, Canada; 2 Bloorview Research Institute, Toronto, Ontario, Canada; 3 Rehabilitation Sciences Institute, University of Toronto, Toronto, Ontario, Canada; 4 Department of Psychology, University of Calgary, Calgary, Alberta, Canada; 5 Neuroscience and Mental Health Program, Hospital for Sick Children, Toronto, Ontario, Canada; 6 Department of Physiology, University of Toronto, Toronto, Ontario, Canada; 7 Neuropsychiatry Program, Department of Psychiatry, Sunnybrook Health Sciences Centre, University of Toronto, Toronto, Ontario, Canada; 8 Division of Neurology, Department of Medicine, Sunnybrook Health Sciences Centre, University of Toronto, Toronto, Ontario, Canada; 9 Brain Sciences Research Program, Sunnybrook Research Institute, Toronto, Ontario, Canada; 10 Department of Pediatrics, University of Toronto, Toronto, Ontario, Canada

## Abstract

**Importance:**

Approximately 15% to 30% of individuals with a history of concussion present with persistent postconcussion symptoms (PPCS). Individuals with PPCS are at greater risk of experiencing depressive symptoms.

**Objective:**

To synthesize the association between depressive symptoms and PPCS in children, adolescents, and adults via meta-analysis and to investigate potential moderators of that association.

**Data Sources:**

Systematic search of Ovid Medline, CINAHL, PsycInfo, and Embase from 1995 to January 2022 was performed. Additionally, references from included studies were hand-searched to ensure relevant articles were captured in the search.

**Study Selection:**

Studies that involved participants who experienced PPCS and quantified depressive symptoms were included. The definition of PPCS was limited to physician-diagnosed or self-reported concussion, with symptoms lasting for a minimum of 4 weeks postinjury. Two authors independently screened all articles to determine study eligibility.

**Data Extraction and Synthesis:**

Study characteristics were extracted independently by 2 trained investigators. Study data were meta-analyzed using a random-effects meta-analysis.

**Exposure:**

PPCS.

**Main Outcomes and Measures:**

The the primary outcome was depressive symptoms.

**Results:**

Data were extracted from 18 studies with a total of 9101 participants. Of the 18 studies, all were cohort studies, and 13 (72%) comprised adult populations. The mean (SD) time since concussion was 21.3 (18.7) weeks. After accounting for potential publication bias, the random-effects meta-analysis found a significant positive association between PPCS and depressive symptoms, (odds ratio, 4.56; 95% CI, 2.82-7.37; *P* < .001). There were no significant moderators, likely due to the small number of studies included.

**Conclusions and Relevance:**

In this meta-analysis, experiencing PPCS was associated with a higher risk of experiencing depressive symptoms. There are several important clinical and health policy implications of the findings. Most notably, the development of strategies for effective prevention and earlier intervention to optimize mental health recovery following a concussion should be supported.

## Key Points

**Question:**

Is prolonged recovery from concussion (via persistent postconcussion symptoms [PPCS]) associated with depressive symptoms postinjury?

**Findings:**

This systematic review and meta-analysis of 18 studies and 9101 participants found that the odds of depressive symptoms were increased 4-fold among individuals who experienced PPCS.

**Meaning:**

These findings suggest that individuals with PPCS are at risk of experiencing symptoms of depression; postinjury support and interventions targeting mental health are necessary for optimal outcomes.

## Teaser

This systematic review and meta-analysis assesses the association between persistent concussion symptoms and depressive symptoms and investigates potential moderators of that association.

## Introduction

Although the rate of concussion (or mild traumatic brain injury [mTBI]) recovery can vary considerably from one individual to another, the vast majority fully recover within days to weeks following the injury.^1,2^ Yet, an important minority (ie, 15%-30%)^3,4^ of individuals who experienced a concussion will experience symptoms for months or years following the injury,^5,6^ often referred to as persistent postconcussion symptoms (PPCS). Although symptoms vary from person to person, headaches, fatigue, dizziness, cognitive difficulties, and emotional changes are commonly reported and can significantly impact an individual’s everyday functioning.^7,8^ PPCS have been associated with a wide range of adverse outcomes, including reduced quality of life, as well as higher levels of disability and psychological distress.^9,10^ The etiology of PPCS is diverse and multifactorial, with prognosis varying between individuals.^2^ The treatment of PPCS remains inconclusive, given that symptoms following a concussion (eg, headache, irritability) are nonspecific and can be commonly reported in healthy individuals, as well as in clinical groups with health conditions unrelated to mTBI.^11,12^ To account for the complexity of PPCS presentation, reference standard guidelines for pediatric and adult populations recommend a comprehensive, interdisciplinary approach to treatment.^13,14^

Throughout the last decade, the association between PPCS and mental health outcomes has emerged as an area of interest. Multiple studies have found bidirectional associations between depressive symptoms and PPCS. Indeed, while individuals with PPCS are significantly more at risk of experiencing depressive symptoms,^15,16^ even for decades following the injury,^17^ depressive symptoms have also been found to predict prolonged recovery following a concussion^9,18^ and are linked to a lower quality of life in individuals with PPCS.^19^ Variables, such as age (older adolescents and younger adults), female sex, mental health history, and the number of previous concussions, have been associated with the development of PPCS.^2,5,20^ Similarly, these variables have also been identified as moderators for the association between PPCS and depressive symptoms.^21,22^

A recent scoping review^23^ highlighted the association between depressive symptoms and mTBI in pediatric populations. Building from these findings, our team also recently published a scoping review broadly exploring the mental health outcomes of individuals with PPCS.^24^ Most studies (76%) included in this review focused on adult populations and mental health symptoms, such as anxiety or depression. Across studies, individuals with PPCS had greater mental health difficulties than individuals who recovered from concussion or healthy controls. Although this scoping review was informative and provided a thorough overview of current research within the field, quantitative summaries evaluating the magnitude and nature of the association between PPCS and mental health outcomes were not conducted. Therefore, it was concluded that a follow-up meta-analysis was warranted to assess the strength and robustness of evidence reported in the studies and provide a more precise estimate of the association.

Consequently, we completed a meta-analysis for this study examining the association between depressive symptoms and PPCS. To our knowledge, the hypothesis that PPCS may be associated with depressive symptoms has not been corroborated in meta-analyses. Based on past literature, we expected to find a strong association between PPCS and depressive symptoms; wherein more PPCS would be associated with more depressive symptoms. A second aim was to investigate potential moderators of that association and determine whether the association between depressive symptoms and PPCS differed based on age, sex, mental illness, history of concussion, and time since the injury. Such research has significant public health implications as it represents an important step toward understanding the association between PPCS and mental health. In turn, this would allow for the development of optimal postconcussion intervention strategies, targeting effective prevention and earlier intervention to enhance recovery trajectories, improve mental health, and promote well-being following concussion.

## Methods

The search strategy from our previous scoping review^24^ formed the basis for the primary inclusion criteria for this systematic review and meta-analysis. This scoping review sought to investigate mood outcomes, such as anxiety and depression, as well as other psychological factors (eg, irritability and aggression) that have been associated with PPCS. We then developed a set of secondary inclusion criteria to ensure more precise study selection, in keeping with the goals of this systematic review and meta-analysis. This systematic review and meta-analyses followed the Preferred Reporting Items for Systematic Reviews and Meta-analyses (PRISMA) reporting guideline.^25^

### Search Strategy

A review protocol was established before beginning the literature search (eAppendix in Supplement 1). Our primary search terms^24^ included: *PPCS*, *depression*, *anxiety*, *concussion*, *mild TBI*, and *mTBI*. The terms *PPCS* and *PCS*, or any combination of *chronic*, *long-term*, *persistent* or *prolonged* and *mTBI*, *mild TBI*, *mild brain injury*, *concussion*, *minor TBI* or *mild/minor head injury* were used to capture the various PPCS terminology in the literature. PPCS was defined herein as postconcussion symptoms lasting at least 4 weeks.^25,26,27^ The search query was composed of subject headings and keywords in: CINAHL (EBSCOhost), PsycINFO, MEDLINE (Ovid), and EMBASE.

### Inclusion and Exclusion Criteria

#### Broad Primary Criteria

To be eligible for the current meta-analysis, all articles from the search framework were screened broadly for PPCS criteria. To qualify, study participants must have experienced a concussion as diagnosed by a health care professional or as classified by diagnostic measures (eg, *International Statistical Classification of Diseases and Related Health Problems, Tenth Revision *or *Diagnostic and Statistical Manual of Mental Disorders* (Fifth Edition) criteria), Glasgow Coma Scale score between 13 and 15, loss of consciousness 30 minutes or less, and/or posttraumatic amnesia of less than 24 hours. Concussion determined by self-report measures were included if definition criteria were met. Study participants were required to experience at least 1 concussion symptom as classified by a concussion assessment measure (eg, Post-Concussion Symptom Inventory, Post-Concussion Symptom Scale, Sport Concussion Assessment Tool) lasting more than 4 weeks. There was no explicit upper limit on duration. All ages were eligible. Studies including participants in the acute stage of concussion (ie, less than 4 weeks postinjury), moderate-to-severe TBI, or unrelated head injuries (eg, edema, fractures, hemorrhages) were excluded.

All study designs were included, aside from opinion articles, editorials, dissertations, and reviews. The searches were restricted to peer-reviewed English-language publications or translations. Studies were eligible if published from 1995 onwards, given that PPCS terminology emerged during that time frame.^28^

#### Secondary Criteria

All studies that passed our broad primary criteria went through a second round of full-text screening to isolate studies measuring depressive symptoms. We defined depressive symptoms as an outcome that must be measured by a validated and standardized measure of depression. Depressive symptoms were examined along a spectrum; whether symptoms amounted to a clinical diagnosis of depression or not was not taken into consideration. Studies that examined anxiety symptoms or other mental health outcomes exclusively were not included in the meta-analysis. Studies that examined depressive symptoms in addition to other mental health outcomes could be included, provided they had distinct measures and results for depressive symptoms.

### Study Selection

For this systematic review and meta-analysis, a search was performed in January 2022. Two independent reviewers searched the 4 databases to synthesize the literature. In total, 13 025 articles were transferred to Covidence for the screening processes. Title and abstract screening were conducted first, followed by full-text screening. All reviewers followed clear, pre-established inclusion and exclusion criteria (presented above). Periodically, the 2 reviewers met to discuss any screening conflicts to resolve them in a timely manner. Studies were retained when consensus was reached.

**Figure 1.**
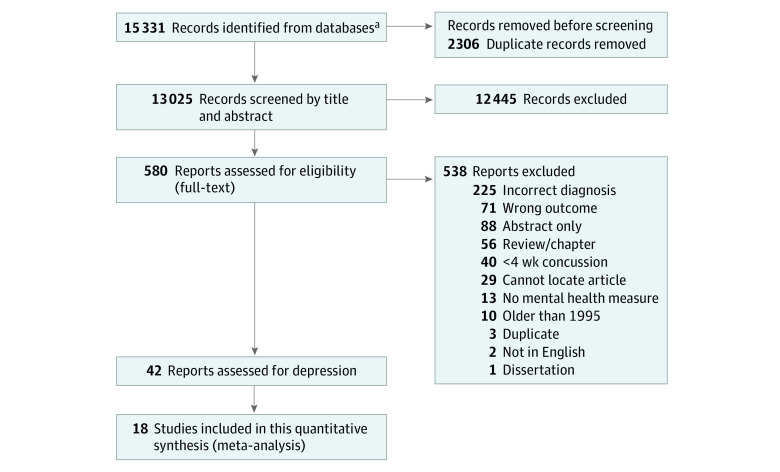
Flow Diagram of Study Selection ^a^Total number accounts for original scoping review search (March 2021) and updated search for systematic review (March 2022) using the same search queries.

Following title and abstract screening, 580 articles advanced to full-text screening. Following full-text screening of broad primary criteria, 42 articles met inclusion. The secondary inclusion criteria (ie, inclusion of a depression measure) was then applied to these 42 articles. Overall, 19 studies met inclusion; 23 studies solely included an anxiety or mental health measure other than depression and were therefore excluded. Of the 19 studies that met secondary inclusion criteria, only 18 studies were included in the analyses and synthesis for this systematic review and meta-analysis (Table 1); 1 study was excluded because it lacked necessary quantitative data.^44^ See Figure 1 for full PRISMA diagram, along with exclusion justification.

**Table 1.** Sample Characteristics for Studies Included in the Meta-analysis

Study	No.	Location	Pediatric sample	Mean (SD) age, y	Gender		History of ≥2 concussions (% sample)	Weeks since concussion	History of mental illness (% sample)	PPCS assessment	Depressive Sx assessment
					Men, No. (%)	Women, No. (%)					
Bunt et al,^29^ 2021	332	United States	Yes	15.1	179 (53.9)	153 (46.1)	30.7	12.0	11.7	SCAT	PHQ
Donnell et al,^30^ 2012	4462	United States	No	37.8	4462 (100)	0 (0)	NA	NA	NA	MHQ	DIS
Eman Abdulle et al,^31^ 2020	162	Netherlands	No	71.6	89 (55.0)	73 (45.0)	NA	24.0	4.0	HISC	HADS
Faulkner et al,^32^ 2021	169	New Zealand	No	35.2	58 (34.3)	111 (65.7)	43.2	8.7	49.1	RPQ	DASS
Lange et al,^33^ 2015	72	Canada	No	34.1	52 (66.9)	20 (33.1)	NA	6.7	NA	BC-PSI	BDI
Lange et al,^22^ 2011	190	Canada	No	36.3	83 (43.7)	107 (56.3)	22	7.8	NA	BC-PSI	BC-MDI
Levin et al,^20^ 2021	2299	United States	No	41.6	1530 (43.7)	769 (56.3)	28.6	5.4	14.8	RPQ	BSI, PHQ
Mooney et al,^34^ 2005	67	United States	No	41.4	37 (55.2)	30 (44.8)	25	60.0	54.0	PCSC	BDI
Morissette et al,^35^ 2011	213	United States	No	38	185 (87.0)	28 (13.0)	NA	NA	NA	BTBIS	BDI
Oldenburg et al,^36^ 2018	94	Sweden	No	39.3	57 (60.6)	37 (39.4)	NA	52.0	NA	RPQ	HADS
Popov et al,^37^ 2021	125	Canada	No	37.7	44 (35.2)	81 (64.8)	54.6	56.7	41.6	RPQ	PHQ
Rapoport et al,^38^ 2003	152	Canada	No	44.2	111 (65.3)	59 (34.7)	23	7.4	29.3	RPQ	GHQ
Rieger et al,^39^ 2019	48	United States	Yes	14.9	22 (45.8)	26 (54.2)	41.7	8.9	4.2	ImPACT	BASC
Stazyk et al,^40^ 2017	90	Canada	Yes	15.0	36 (39.1)	56 (60.9)	45.5	21.9	3.3	PCSI	CDI
Stein et al,^41^ 2017	49	United States	Yes	15.0	17 (34.7)	32 (65.3)	52.1	19.4	18.0	HBI	PHQ
Waljas et al,^2^ 2015	124	Finland	No	38.2	56 (44.4)	70 (55.6)	34.9	28.0	7.1	RPQ	BDI
Wright et al,^42^ 2021	298	United States	Yes	15.1	142 (47.7)	156 (52.3)	32.2	13.4	12.8	SCAT	PHQ
Yang et al,^43^ 2007	155	China	No	35.8	75 (48.5)	80 (51.5)	NA	8.0	NA	CPCS	GOSE

Abbreviations: BASC, Behavior Assessment System for Children; BC-MDI, British Columbia Major Depression Inventory; BC-PSI, British Columbia Postconcussion Symptom Inventory; BDI, Beck Depression Inventory; BSI, Brief Symptom Inventory; BTBIS, Brief Traumatic Brain Injury Screen; CDI, Children’s Depression Inventory; CPCS, Checklist of Postconcussion Symptoms; DASS, Depression, Anxiety and Stress Scale; DIS, Diagnostic Interview Schedule; GHQ, General Health Questionnaire; GOSE, Glasgow Outcome Scale-Extended; HADS, Hospital Anxiety and Depression Scale; HBI, Health Behavior Inventory; ImPACT, Immediate Postconcussion Assessment and Cognitive Testing; HISC, Head Injury Symptom Checklist; MHQ, medical history questionnaire; NA, not applicable; PCSC, Post-Concussion Syndrome Checklist; PCSI, Post-Concussion Symptom Inventory; PHQ, Patient Health Questionnaire; RPQ, Rivermead Post-Concussion Symptoms Questionnaire; SCAT, Sport Concussion Assessment Tool.

### Data Extraction and Synthesis

Data extraction was charted using a Microsoft Excel spreadsheet, co-created by reviewers to document all relevant details of each study. General study characteristics (eg, title, author, year of publication, geographical location) along with several potential moderator variables (eg, sex, age, time since injury, preexisting mental illness, prior concussion history, mechanism of injury) were extracted from individual studies. The relevant statistics regarding the association between PPCS and depressive symptoms were recorded and analyzed.

### Quality Assessment

We assessed the quality of evidence using the 5 domains of the GRADE guidelines^45^: risk of bias, imprecision, inconsistency, indirectness, and publication bias. As suggested by the GRADE, the risk of bias was assessed using the Newcastle-Ottawa Quality Assessment Scale Cohort Studies (eTable 1 and eTable 2 in Supplement 1).

### Statistical Analysis

Studies reported on the association between PPCS and depressive symptoms using different formats of effect sizes (eg, odds ratios [ORs], correlations, regression coefficients). Effect sizes were converted to ORs using standard effect conversion formulas in order to convert all data to a common scale for meta-analysis.^46^ Some studies reported on multiple effect sizes (eg, 2 effect sizes representing different measures of depression). In such cases, we used metafor^47^ to pool across effect sizes to ensure that each study was only represented by 1 effect size. The random-effects meta-analysis was also conducted using the R package metafor version 3.0.2 (R Project for Statistical Computing). The analysis relied on converted log OR values, which are converted back to odd ratios for ease of interpretation.^48^ Consistent with the Cohen guidelines^49^ for the interpretation of effect sizes, where ORs of 1.44, 2.48, and 4.27 were considered small, medium, and large in magnitude, respectively. Level of statistical significance was set to *P* < .05. Publication bias was examined via Egger test and the funnel plots. Lastly, the presence of heterogeneity was assessed using Cochrane Q and I^2^ statistics. A significant Q suggests that the analysis of moderators is warranted, while an I^2^ value greater than 75% suggests the presence of considerable heterogeneity.^50^

## Results

### Sample Characteristics

Eighteen studies reported on the association between PPCS and depressive symptoms, and included a total of 9101 participants. The median (range) sample size was 154 (48-4462) participants.^30,39^ The mean (SD) participant age was 33.7 (14.4) years old (median [range], 37 [14.9-56.7] years). A mean (SD) of 36.1% (11.1%) of participants had a history of 2 or more concussions (median [range], 33.6% [22%-54.6%]). The mean (SD) length of time since the concussion was 21.3 (18.7) weeks (median [range], 12.7 [5.4-60.0] weeks). Most studies (13 [72%]) used a cross-sectional design. Publication year ranged from 2003 to 2021. With respect to geographic location, most studies were conducted in North America (Canada, 5 [27.8%]^22,33,37,38,40^; United States, 8 [44.4%]^20,29,30,34,35,39,41,42^), with the rest conducted in Europe (Finland, 1 [5.6%]^2^; Netherlands, 1 [5.6%]^31^; Sweden, 1 [5.6%]),^36^ China (1 [6%]),^43^ and New Zealand (1 [5.6%]).^32^ The most frequently used questionnaires to assess PPCS was the Rivermead Post-Concussion Symptoms Questionnaire (6 [33.3%]).^2,20,32,36,37,38^ The most frequently used questionnaires to assess depressive symptoms included the Patient Health Questionnaire (5 [27.8%]),^20,29,37,41,42^ the Beck Depression Inventory-II (4 [22.2%]),^2,33,34,35^ and the Hospital Anxiety and Depression Scale (2 [22.2%])^31,36^ (Table 1).

### Meta-Analytical Results

**Figure 2.**
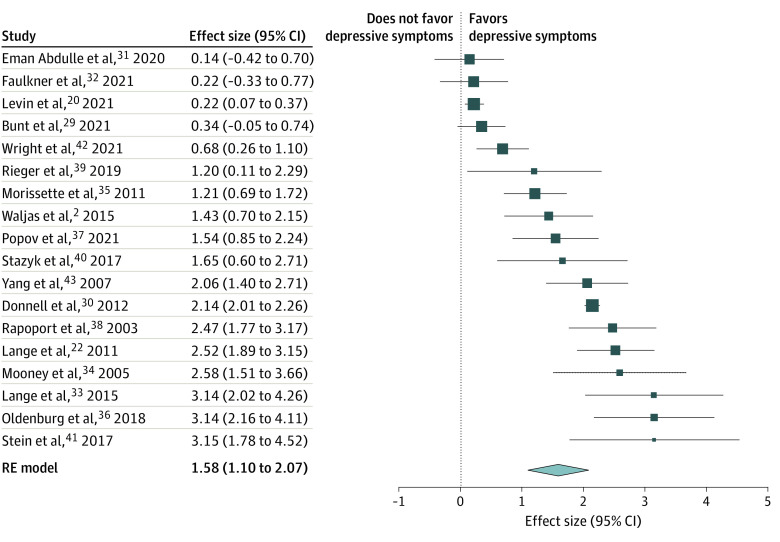
Forest Plot of Effect Sizes for the Association Between Persistent Postconcussion Symptoms and Depressive Symptoms The effect sizes are presented from the smallest to the largest effect size. The effect sizes were analyzed and presented as log odds ratio. RE indicates random-effects. The size of the boxes indicates the sample size of the study.

The random-effects meta-analysis identified a significant positive association between PPCS and postinjury depressive symptoms (OR, 4.87; 95% CI, 3.01-7.90; *P* < .001), representing a large effect size (Figure 2).^49^ The funnel plot (eFigure 1 in Supplement 1) and Egger test (*z* = 2.84; *P* = .005) suggested the presence of potential publication bias. Tweedie and Duval trim-and-fill method was used to evaluate the extent of the potential bias. Results indicated that 5 studies were missing on the left side (eFigure 2 in Supplement 1). Even after accounting for this potential publication bias, the effect size was of large magnitude (OR, 4.56; 95% CI, 2.82-7.37; *P* < .001). Although there was high heterogeneity between studies (*I^2^* = 95.74%; *Q* = 508.03, *P* < .001), the moderator analysis failed to identify significant moderators (Table 2 and Table 3). The GRADE ratings for the body of literature synthesized are available in the eTable 3 in Supplement 1, and showed low quality primarily due to the use of self-reported measures and the lack of randomized control trials.

**Table 2.** Results of Categorical Moderator Analysis

Categorial moderators	No. of Studies	OR (95% CI)	*P* value of the effect size	Q_M_	*P* value of the moderation test
Geographic location					
North America	13	5.39 (3.01 to 9.63)	<.001	0.38	.54
Outside of North America	5	3.83 (1.52 to 9.66)	.005		
Measure of depression					
Beck Depression Index	4	7.49 (2.69 to 20.9)	<.001	2.29	.32
Patient Health Questionnaire	5	2.82 (1.17 to 6.81)	.02		
Other measures	9	5.53 (2.84 to 10.76)	<.001		
Measure of PPCS					
Rivermead	6	4.23 (1.82 to 9.84)	<.001	0.18	.68
Other measures	12	5.28 (2.87 to 9.73)	<.001		
Pediatric sample					
Yes	5	3.59 (1.39 to 9.27)	.008	0.55	.46
No	13	5.45 (3.09 to 9.60)	<.001		

Abbreviations: OR, odds ratio; PPCS, persistent postconcussion symptoms; Q_M_, test of moderator.

**Table 3.** Results of Continuous Moderator Analysis

Continuous moderators	No. of studies	B (95% CI)	Z	*P* value
Age, y	18	−0.005 (−0.04 to 0.03)	−0.28	.78
Gender (% men)	18	0.02 (−0.01 to 0.04)	1.05	.30
History of ≥2 concussions (% of sample)	12	−0.01 (−0.06 to 0.05)	−0.17	.87
History of mental illness (% of sample)	12	0.02 (−0.02 to 0.05)	0.91	.37
Time since concussion, wk	16	0.07 (−0.01 to 0.05)	1.10	.27

## Discussion

This systematic review and meta-analysis provides a current and comprehensive examination of depressive symptoms in individuals experiencing PPCS. These results show an association between PPCS and depressive symptoms. Over the years, several research teams have aimed to gain more insight into the etiology and underlying mechanisms of development and course of mental health difficulties in individuals who experience a concussion. Given the myriad of contributing physiological, biological, and psychosocial factors, a biopsychosocial framework has often been used to examine the relationship between PPCS and depressive symptoms.^51^ While some studies have highlighted the role of the trigeminal cervical complex and pain neuropeptides in specific PPCS, such as headache,^52^ depressive symptoms following a concussion could be a direct consequence of postinjury brain changes in the autonomic nervous system, more specifically the amygdala, a brain structure strongly connected to autonomic and neurohormonal axes that plays a crucial role in emotional processing.^53,54,55^ From a psychosocial standpoint, individuals experiencing PPCS often face increased social isolation, academic difficulties, frustration surrounding activity limitations, anxiety related to prognosis uncertainties, and prolonged disengagement from usual roles and identity structures—all factors that may contribute to feelings of hopelessness and emotional distress.^56,57,58^ These neurofunctional and psychosocial underpinnings of depressive symptoms in individuals with PPCS may interact with one another. For instance, emotional processing plays a central role for successful social interactions and interpersonal relationships.^59,60^ Changes in structures essential for emotional processing, such as the amygdala, may therefore have bidirectional interconnections with social difficulties and isolation.

While the results of the current study highlight the strong magnitude of the association between PPCS and postinjury depressive symptoms, it does not allow inference about the causal directionality of the association. The question remains—do PPCS induce depressive symptoms, or do depressive symptoms induce PPCS? Although the bidirectional and interdependent nature of the relationship and the contribution of multifactorial biological and psychosocial factors are undeniable, it could be argued that studies examining the treatment of psychiatric difficulties following TBI provide insight into the directionality of the association. Several randomized clinical trials show that treating depressive symptoms following TBI leads to improvement in mental health outcomes, somatic and cognitive symptoms and overall functioning.^61^ Depressive symptoms may magnify the burden of concussion symptoms and lead to PPCS. The proactive detection and management of depressive symptoms following a concussion may potentially be an effective prevention for PPCS. Longitudinal studies are essential to gain further insight into temporal patterns and directionality of the association.

This review did not identify significant moderators of the association between PPCS and depressive symptoms. This is likely explained by the small number of included studies, the limited information provided by each article, and the lack of sample diversity. The small number of studies is a considerable limitation to reliably assess moderators of the association and evaluate the effects of additional variables, such as the mechanism of injury. Future studies should report sample characteristics and whether associations differ based on those characteristics. Moderators may emerge as more data becomes available. The reliable identification of moderators would offer opportunities to identify subpopulations at greater risk of poor mental health outcomes following a concussion and inform the development of individualized approaches to concussion care and management rather than applying a one-size-fits-all model.^62^ It would also allow for the development of screening interventions for specific pre-injury characteristics and more effective strategies for optimal care.

### Clinical Implications

Our findings may provide a future reference for the role of depressive symptoms in concussion prognosis and have significant clinical implications and public health importance. The results of this review not only highlight the need for a greater understanding of the mechanisms of development and etiology of depressive symptoms postconcussion, but also emphasize the necessary emergence for timely and effective treatment interventions for depressive symptoms to optimize the long-term prognosis of concussion. Our results also suggest that specialized multidisciplinary or interdisciplinary concussion care programs should include health care professionals with strong clinical foundations and training in mental health conditions (eg, psychologists, social workers, psychiatrists). Additional studies on the management of postconcussion mental health difficulties are of utmost importance, given the strong association between concussion and suicidality.^63^ Unfortunately, very limited guidelines are currently available for the management of depressive symptoms following a concussion.^62^ Efficacy of intervention modalities should be investigated in future studies.

### Limitations

This review had limitations. The heterogeneity in methods used to study PPCS impacted the data extracted and analyzed in the current review. The body of evidence contained limitations in terms of quality (eg, self-reported measures, no RCTs). There was inconsistent operationalization of PPCS across studies which may have introduced some bias in the results. Although most of the studies did not use a reference standard diagnostic interview to identify mental health status, they all used validated self-report measures, which are acceptable methods in mental health research. This review did not determine the directionality of the relationship between PPCS and depressive symptoms, and further research is required to make any causal inference.

## Conclusions

This systematic review and meta-analysis provided estimates of the association between PPCS and depressive symptoms. We found a positive association between PPCS and depressive symptoms, and these findings support the need for mental health interventions in concussion rehabilitation. Gaining further knowledge on PPCS and identifying target variables that improve long-term outcomes are critical to inform the development of optimal concussion care plans. With the high annual prevalence rates, concussions must remain a priority in clinical research, and efforts to better understand, monitor, and mitigate their adverse long-term consequences are needed. Future studies should attempt to expand the understanding of underlying mechanisms that connect PPCS and depressive symptoms and evaluate the efficacy of different preventative strategies.

## Notes

Supplement 1. **eAppendix.** Complete database search strategy framework **eFigure 1.** Funnel plot of the association between PPCS and depressive symptoms **eFigure 2. **Funnel plot with Tweedie and Duval trim-and-fill method **eTable 1. **Description of the risk of bias scale **eTable 2. **Study Scores on the Newcastle-Ottawa Quality Assessment Scale **eTable 3.** Evaluation of the quality of research Using the GRADE Guidelines

Supplement 2.
Data Sharing Statement
