# Supplementary material for: Depressive Symptoms in Individuals With Persistent Postconcussion Symptoms: A Systematic Review and Meta-Analysis
Source: JAMA Netw Open. 2022 Dec 27;5(12):e2248453. doi: 10.1001/jamanetworkopen.2022.48453 (PMC9857135; doi:10.1001/jamanetworkopen.2022.48453)
Supplement: Supplement 1. — eAppendix. Complete database search strategy framework eFigure 1. Funnel plot of the association between PPCS and depressive symptoms eFigure 2. Funnel plot with Tweedie and Duval trim-and-fill method eTable 1. Description of the risk of bias scale eTable 2. Study Scores on the Newcastle-Ottawa Quality Assessment Scale eTable 3. Evaluation of the quality of research Using the GRADE Guidelines [file jamanetwopen-e2248453-s001.pdf]

Supplemental Online Content

Lambert M, Sheldrake E, Deneault AA, Wheeler A, Burke M, Scratch S. Depressive symptoms in individuals with persistent postconcussion symptoms: a systematic review and meta-analysis. *JAMA Netw Open*. 2022;5(12):e2248453. doi:10.1001/jamanetworkopen.2022.48453

eAppendix 1. Complete database search strategy framework
eFigure 1. Funnel plot of the association between PPCS and depressive symptoms
eFigure 2. Funnel plot with Tweedie and Duval trim-and-fill method
eTable 1. Description of the risk of bias scale
eTable 2. Study Scores on the Newcastle-Ottawa Quality Assessment Scale
eTable 3. Evaluation of the Quality of Research Using the GRADE Guidelines

This supplemental material has been provided by the authors to give readers additional information about their work.

© 2022 Lambert M et al. *JAMA Network Open.*

eAppendix. Complete database search strategy framework

Ovid MEDLINE Search Outline

#	Search Query
1	post-concussion syndrome/
2	brain injuries, traumatic/ or brain concussion/ or brain injury, chronic/
3	(post-concuss* or post concuss* or postconcuss).tw,kf
4	(mtbi or mild trauma* brain injur* or mild TBI).tw,kf
5	((persist* or prolonged or chronic or long-term) adj3 (brain injur* or concuss* or head injur*)).tw,kf.
6	1 or 2 or 3 or 4 or 5
7	mood disorders/ or mental health/ or depressive disorder, major/ or anxiety disorder/ or depressive disorder/
8	((mood or major depress* or anxi* or generalized anxiety*) adj3 (disorder* or syndrome* or outcome*)).tw,kf
9	emotions/ or anger/ or anxiety/ or psychological distress/ or frustration/ or hostility/ or sadness/ or confusion/ or irritable mood/ or impulsive behaviour
10	(emotion* or irritable or irritability or angry or anger or anxi* or distress* or frustrat* or hostil* or sad* or confus* or worr* or impulsiv* or attention*).tw,kf
11	7 or 8 or 9 or 10
12	6 and 11

PsychInfo Search Outline

#	Search Query
1	brain injuries, traumatic/ or brain concussion/ or brain injury, chronic/
2	(post-concuss* or post concuss* or postconcuss).tw
3	(mtbi or mild trauma* brain injur* or mild TBI).tw
4	((persist* or prolonged or chronic or long-term) adj3 (brain injur* or concuss* or head injur*)).tw
5	post-concussion syndrome.tw

© 2022 Lambert M et al. *JAMA Network Open.*

6	mood disorders/ or mental health/ or depressive disorder, major/ or anxiety disorder/ or depressive disorder/
7	((mood or major depress* or anxi* or generalized anxiety*) adj3 (disorder* or syndrome* or outcome*)).tw
8	emotions/ or anger/ or anxiety/ or psychological distress/ or frustration/ or hostility/ or sadness/ or confusion/ or irritable mood/ or impulsive behaviour
9	(emotion* or irritable or irritability or angry or anger or anxi* or distress* or frustrat* or hostil* or sad* or confus* or worr* or impulsiv* or attention*).tw
10	1 or 2 or 3 or 4 or 5
11	6 or 7 or 8 or 9
12	affective disorders/
13	acting out/
14	11 or 12 or 13
15	10 and 14

CINAHL Search Outline

#	Search Query
1	(MH "Postconcussion Syndrome")
2	(MH "Brain Concussion+")
3	T1 (mild TBI or mTBI or mild trauma* brain injur*) OR AB (mild TBI or mTBI or mild trauma* brain injur*)
4	T1 (post-concuss* or postconcuss* or post concuss*) OR AB (post-concuss* or postconcuss* or post concuss*)
5	T1 (chronic brain injur* OR AB chronic brain injur*)
6	T1 ((persist* or prolonged or chronic or long-term) N3 (brain injur* or concuss* or head injur*) OR AB ((persist* or prolonged or chronic or long-term) N3 (brain injur* or concuss* or head injur*))
7	(MH "Anxiety+") OR (MH "Anxiety Disorders+") OR (MH "Generalized Anxiety Disorder")
8	(MH "Depression+")
9	(MH "Affective Disorders+")

© 2022 Lambert M et al. *JAMA Network Open.*

10	(MH “Mental Health”)
11	T1 ((mood or mental or affective or anxiet* or depress*) N3 (disorder* or syndrome* or outcome*)) OR AB ((mood or mental or affective or anxiet* or depress*) N3 (disorder* or syndrome* or outcome*))
12	(MH “Emotions+”)
13	(MH “Sadness”)
14	(MH “Confusion”)
15	(MH “Anger”)
16	(MH “Acting Out”)
17	(MH “Attention+”) OR (MH “Attention Deficit Hyperactivity Disorder”)
18	T1 (emotion* or irritab* or anger or angry or sad* or distress* or frustrat* or confus* or worr* or impuls* or attention or hostil*) OR AB (emotion* or irritab* or anger or angry or sad* or distress* or frustrat* or confus* or worr* or impuls* or attention or hostil*)
19	1 or 2 or 3 or 4 or 5 or 6
20	7 or 8 or 9 or 10 or 11 or 12 or 13 or 14 or 15 or 16 or 17 or 18
21	19 and 20

EMBASE Search Outline

#	Search Query
1	brain injuries, traumatic/ or brain concussion/ or brain injury, chronic/
2	(post-concuss* or post concuss* or postconcuss).tw,kw
3	(mtbi or mild trauma* brain injur* or mild TBI).tw,kw
4	((persist* or prolonged or chronic or long-term) adj3 (brain injur* or concuss* or head injur*)).tw,kw
5	exp postconcussion syndrome/
6	1 or 2 or 3 or 4 or 5
7	mood disorders/ or mental health/ or depressive disorder, major/ or anxiety disorder/ or depressive disorder/
8	((mood or major depress* or anxi* or generalized anxiety*) adj3 (disorder* or syndrome* or outcome*)).tw,kw

© 2022 Lambert M et al. *JAMA Network Open.*

9	emotions/ or anger/ or anxiety/ or psychological distress/ or frustration/ or hostility/ or sadness/ or confusion/ or irritable mood/ or impulsive behaviour
10	(emotion* or irritable or irritability or angry or anger or anxi* or distress* or frustrat* or hostil* or sad* or confus* or worr* or impulsiv* or attention*).tw,kw
11	7 or 8 or 9 or 10
12	6 and 11

© 2022 Lambert M et al. *JAMA Network Open.*

eFigure 1. Funnel plot of the association between PPCS and depressive symptoms.

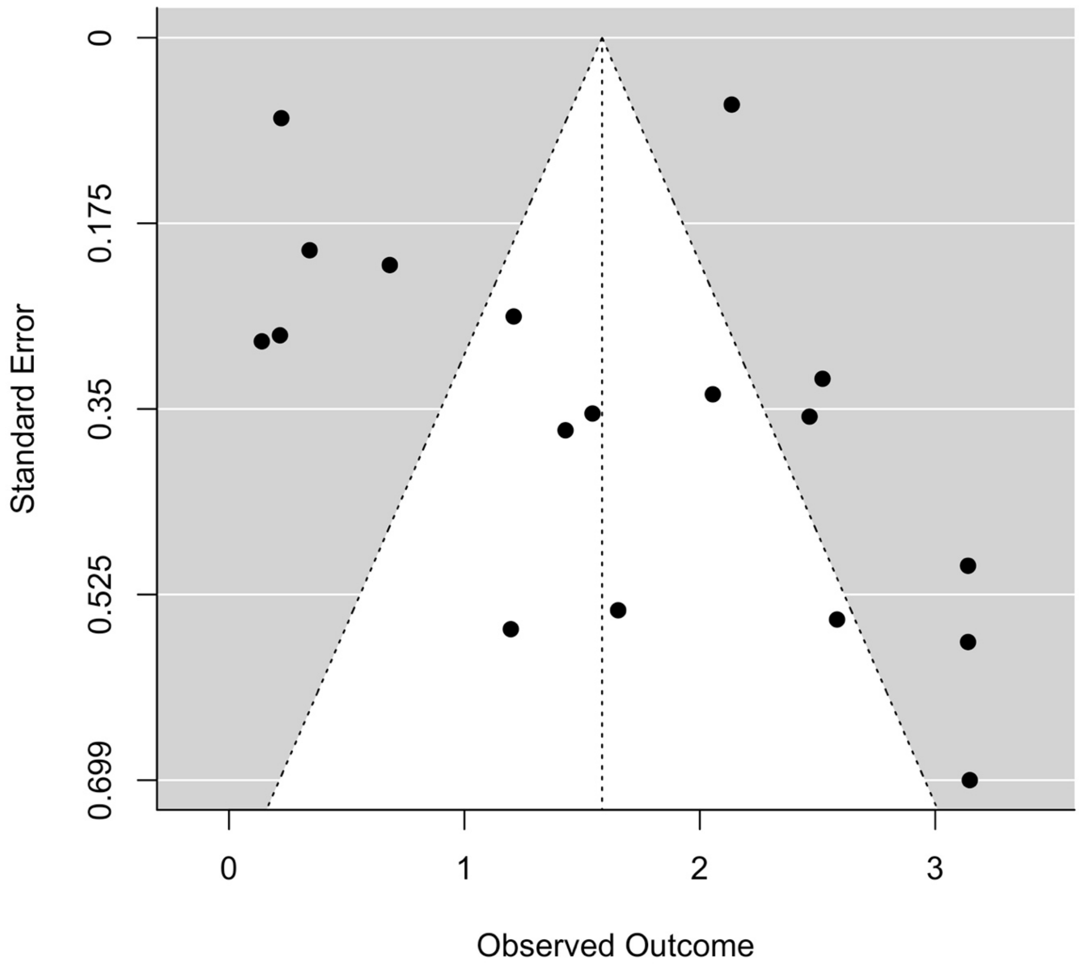

© 2022 Lambert M et al. *JAMA Network Open.*

eFigure 2. Funnel plot of the association between PPCS and depressive symptoms with Tweedie and Duval's trim-and-fill method

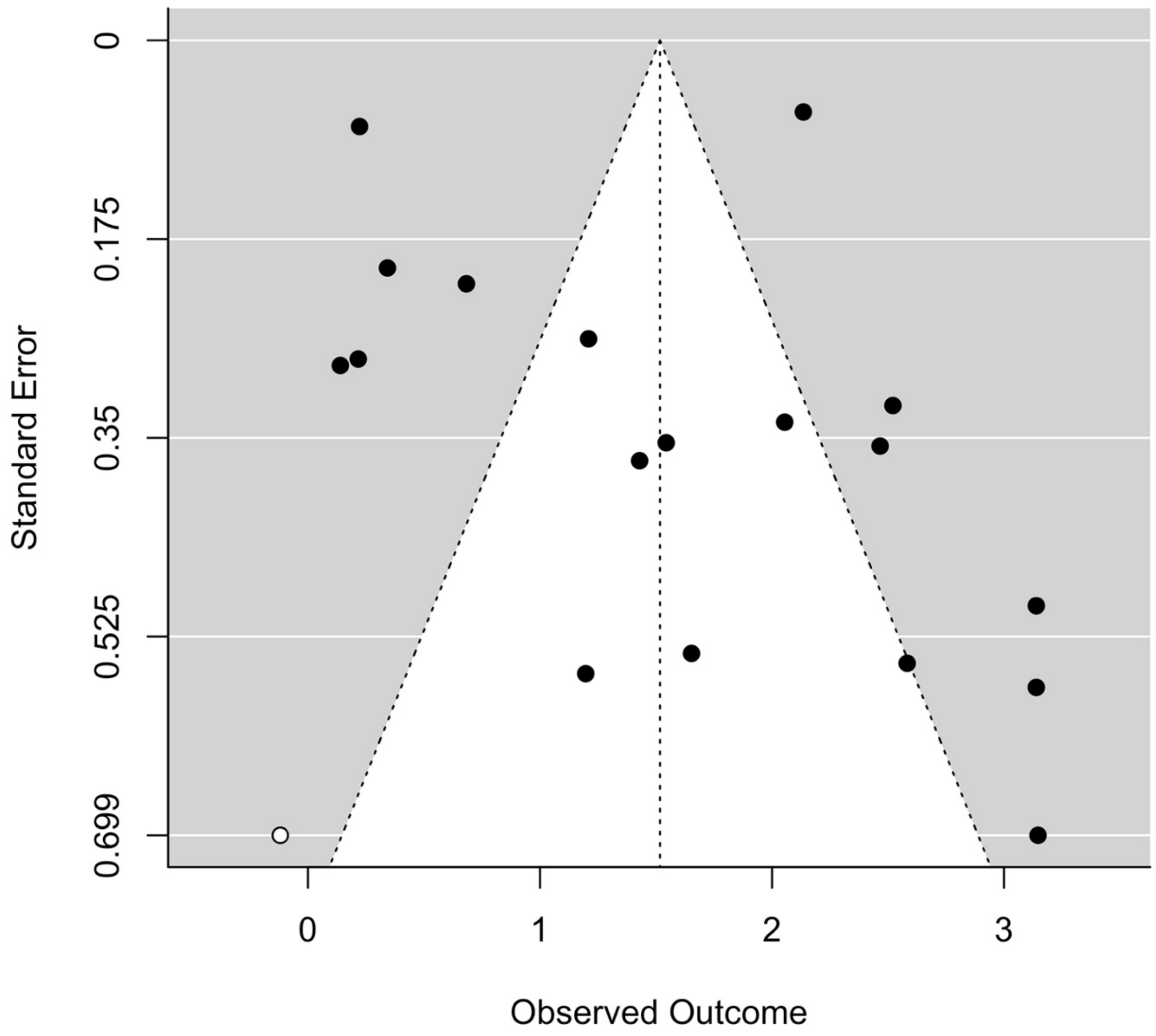

© 2022 Lambert M et al. *JAMA Network Open.*

eTable 1. Risk assessment tool adapted from the Newcastle-Ottawa Quality Assessment Scale Cohort Studies

Selection

1) Representativeness of the mTBI cohort with PPCS
   a) truly representative of the average person who suffers PPCS in the community ✵
   b) somewhat representative of the average person who suffers PPCS in the community (e.g., selected age range, but among age ranges most at risk for PPCS) ✵
   c) selected group of users (e.g., military personnel)
   d) no description of the derivation of the cohort

2) Selection of the non-exposed cohort
   a) drawn from the same community of patients with mTBI (e.g., without PPCS) ✵
   b) drawn from a different source (e.g., community controls)
   c) no description of the derivation of the non-exposed cohort or no non-exposed cohort

3) Ascertainment of PPCS
   a) secure record ✵
   b) structured interview (e.g., with a clinician) ✵
   c) self-report questionnaire
   d) no description

4) Reports if participants previously suffered from depressive symptoms
   a) yes ✵
   b) no

Comparability

1) Comparability of cohorts on the basis of the design or analysis
   a) study considers age ✵
   b) study considers previous mTBI injury ✵

Outcome

1) Assessment of depressive symptoms
   a) independent blind assessment ✵
   b) structured interview with a clinician ✵
   c) self-report questionnaire
   d) no description

2) Was follow-up long enough for outcomes to occur
   a) yes (4 weeks after concussion) ✵
   b) no

3) Adequacy of follow-up of cohorts
   a) complete follow-up - all subjects accounted for ✵
   b) subjects lost to follow up unlikely to introduce bias - small number lost - > 80 % follow up, or description provided of those lost) ✵
   c) follow-up rate < 80% and no description of those lost
   d) no statement describing if participants were lost

© 2022 Lambert M et al. *JAMA Network Open.*

eTable 2. Study Scores on the Newcastle-Ottawa Quality Assessment Scale

Study	Selection	Comparability	Outcome
Bunt, 2021	**	**	
Donnell, 2012	**	*	**
Eman Abdulle, 2020	**	**	
Faulkner, 2021	***	**	*
Lange, 2015	*	*	*
Lange, 2011	*	*	*
Levin, 2021	**	**	**
Mooney, 2005	***	*	*
Morissette, 2011	**	*	
Oldenburg, 2018	***	**	***
Popov, 2021	***	**	**
Rapoport, 2003	***	**	***
Rieger, 2019	***	**	**
Stazyk, 2017	***	**	*
Stein, 2017	***		*
Waljas, 2015	**	**	*
Wright, 2021	***	**	*
Yang, 2007	***	*	*

© 2022 Lambert M et al. *JAMA Network Open.*

eTable 3. Evaluation of the Quality of Research Using the GRADE Guidelines

**Domain**	**Evaluation**
Risk of bias	The quality of studies is generally high. Almost all studies selected a representative cohort of participants, and reported on relevant covariates. The biggest concern is that almost all studies relied on questionnaire-based measures, although this is not uncommon from this literature
Imprecision	Although the confidence intervals vary, their interpretation remains the same across the lower and upper bounds given that individuals with PPCS are 3 to 7 times more likely to experience depressive symptoms. This is an important outcome deserving clinical attention at both the lower and upper possible bounds of the effect size
Inconsistency	The synthesized body of literature shows some heterogeneity. The statistical test for heterogeneity was significant and the I^2 value large. There is, nonetheless, some overlap between confidence intervals and some consistency across point estimates
Indirectness	The studied population is the one for which outcomes are important. The outcomes measures were consistent with the outcomes of interest. Comparisons were inconsistent across studies with respect to the comparison group (or lack thereof)
Publication bias	Publication bias appears limited in this meta-analysis. Study results do not appear to be biased due to industry financing. The publication bias analysis identified only one missing study potentially missing due to publication bias, which did not change the magnitude of the effect size
Overall rating	The GRADE rating for this body of work is low. Given that the studies were not randomized controlled trials for the most part, this results in a default low quality. Nonetheless, the domains do not indicate very serious issues, and quality is marked up for a large magnitude of effect

Abbreviation: PPCS, persistent postconcussion symptoms.

© 2022 Lambert M et al. *JAMA Network Open.*
