# Supplementary material for: Depressive Symptoms in Individuals With Persistent Postconcussion Symptoms: A Systematic Review and Meta-Analysis
Source: JAMA Netw Open. 2022 Dec 27;5(12):e2248453. doi: 10.1001/jamanetworkopen.2022.48453 (PMC9857135; doi:10.1001/jamanetworkopen.2022.48453)
Supplement: Supplement 2. — Data Sharing Statement [file jamanetwopen-e2248453-s002.pdf]

Data Sharing Statement

Lambert. Depressive Symptoms in Individuals With Persistent Postconcussion Symptoms: A Systematic Review and Meta-Analysis. *JAMA Netw Open*. Published December 27, 2022. doi:10.1001/jamanetworkopen.2022.48453

Data
Data available: No
